# O uso de óleos essenciais e aromaterapia no trabalho de parto*

**DOI:** 10.15649/cuidarte.2318

**Published:** 2022-10-20

**Authors:** Gisele Karasek, Júnia Aparecida Laia da Mata, Alessandra Vaccari

**Affiliations:** 1 Hospital Nossa Senhora da Conceigáo, Porto Alegre, Rio Grande do Sul, Brasil. Email: enf.giselekarasek@hotmail.com Hospital Nossa Senhora da Conceição Hospital Nossa Senhora da Conceigáo Porto Alegre Rio Grande do Sul Brazil enf.giselekarasek@hotmail.com; 2 Universidade Federal do Rio Grande do Sul, Porto Alegre, Rio Grande do Sul, Brasil. Email: iumata.2905@qmail.com Universidade Federal do Rio Grande do Sul Universidade Federal do Rio Grande do Sul Porto Alegre Rio Grande do Sul Brazil iumata.2905@qmail.com; 3 Universidade Federal do Rio Grande do Sul, Porto Alegre, Rio Grande do Sul, Brasil. Email: alessandra.vaccari@ufrgs.br Universidade Federal do Rio Grande do Sul Universidade Federal do Rio Grande do Sul Porto Alegre Rio Grande do Sul Brazil alessandra.vaccari@ufrgs.br

**Keywords:** Óleos Voláteis, Aromaterapia, Trabalho de Parto, Essential Oils, Aromatherapy, Labor, Obstetric, Aceites Esenciales, Aromaterapia, Trabajo de Parto

## Abstract

**Introdujo:**

a aromaterapia consiste na utilizacáo de óleos essenciais na prevendo ou no tratamento de diversas afeccóes humanas. No trabalho de parto, pode ser aplicada para o alívio da dor e ansiedade.

**Objetivo:**

identificar na literatura científica sobre o uso da aromaterapia e dos óleos essenciais no manejo do trabalho de parto; e elaborar um protocolo hospitalar, a partir dos achados nas publicares, sobre aromaterapia e aplicado de óleos essenciais no trabalho de parto.

**Materiais e Métodos:**

trata-se de revisáo integrativa da literatura desenvolvida nas bases de dados LILACS, Cochrane Library e Pubmed. Incluíram-se artigos científicos originais publicados no período de 2000 a 2019. O material coletado foi analisado com base na análise temática de conteúdo de Laurence Bardin.

**Resultados:**

treze artigos integraram o corpus desta pesquisa. Deles emergiram quatro categorias de análise: 1- Aromaterapia como estratégia para o alivio da dor na fase de dilatado do trabalho de parto; 2- Aromaterapia como estratégia para a redudo de ansiedade no trabalho de parto; 3- Métodos de administrado dos óleos essenciais no trabalho de parto; e 4- O uso de óleos essenciais para o manejo de sintomas desagradáveis e da progressáo do trabalho de parto.

**Discussao:**

a partir dos resultados, elaborou-se um protocolo hospitalar sobre o uso de aromaterapia no trabalho de parto.

**Conclusao:**

a aromaterapia é uma ferramenta adequada para o cuidado humanizado no manejo da dor e da ansiedade no trabalho de parto, sem efeitos adversos documentados na literatura levantada.

## Abstract (en)

**Introduction:**

aromatherapy consists of the use of essential oils in the prevention or treatment of various human conditions. In labor, it can be applied to relieve pain and anxiety.

**Objective:**

identify in the scientific literature on the use of aromatherapy and essential oils in the management of labor; and to elaborate a hospital protocol, based on the findings in the publications, on aromatherapy and application of essential oils in labor.

**Materials and Methods:**

this is an integrative literature review developed in the LILACS, Cochrane Library and Pubmed databases. Original scientific articles published between 2000 and 2019 were included. The material collected was analyzed based on Laurence Bardin's thematic content analysis.

**Results:**

thirteen articles integrated the corpus of this research. From them, four categories of analysis emerged: 1- Aromatherapy as a strategy for pain relief in the dilatation phase of labor; 2- Aromatherapy as a strategy to reduce anxiety in labor; 3- Methods of administering essential oils in labor; and 4- The use of essential oils for the management of unpleasant symptoms and the progression of labor.

**Discussion:**

based on the results, a hospital protocol was developed on the use of aromatherapy in labor.

**Conclusion:**

aromatherapy is an adequate tool for humanized care in the management of pain and anxiety in labor, with no adverse effects documented in the literature surveyed.

## Resumen

**Introducción:**

la aromaterapia consiste en el uso de aceites esenciales en la prevención o tratamiento de diversas afecciones humanas. En el trabajo de parto, se puede aplicar para aliviar el dolor y la ansiedad.

**Objetivo:**

identificar en la literatura científica sobre el uso de la aromaterapia y los aceites esenciales en el manejo del parto; y elaborar un protocolo hospitalario, basado en los hallazgos de las publicaciones, sobre aromaterapia y aplicación de aceites esenciales en el trabajo de parto.

**Materiales y Métodos:**

se trata de una revisión integrativa de la literatura desarrollada en las bases de datos LILACS, Cochrane Library y Pubmed. Se incluyeron artículos científicos originales publicados entre 2000 y 2019. El material recolectado fue analizado con base en el análisis de contenido temático de Laurence Bardin.

**Resultados:**

trece artículos integraron el corpus de esta investigación. De ellos surgieron cuatro categorías de análisis: 1- La aromaterapia como estrategia para el alivio del dolor en la fase de dilatación del trabajo de parto; 2- La aromaterapia como estrategia para reducir la ansiedad en el parto; 3- Métodos de administración de aceites esenciales en el trabajo de parto; y 4- El uso de aceites esenciales para el manejo de síntomas desagradables y la progresión del trabajo de parto.

**Discusión:**

a partir de los resultados se elaboró un protocolo hospitalario sobre el uso de la aromaterapia en el trabajo de parto.

**Conclusión:**

la aromaterapia es una herramienta adecuada para el cuidado humanizado en el manejo del dolor y la ansiedad en el trabajo de parto, sin efectos adversos documentados en la literatura investigada.

## Introdujo

O processo de parto é reconhecido como um evento fisiológico e natural, vivenciado de maneiras diferentes pelas mulheres^1^. Um estudo recente de Lehugeur^2^, mostrou grande frequéncia na utilizagáo de métodos nao farmacológicos utilizados por enfermeiras obstetras no manejo do trabalho de parto (TP) para alívio da dor e diminuido da ansiedade, tais como massagem, aromaterapia, escalda-pés e musicoterapia. Promover a sensagáo de bem-estar no TP contribui para a satisfagáo materna, além de propiciar uma experiéncia parturitiva positiva.

Há pesquisas no domínio do desenvolvimento humano que sugerem que as patologias ao longo da vida podem comegar com adversidades precoces, como o estresse pré-natal^3^. Os impactos dos eventos estressores no desenvolvimento do hipocampo demonstram como é prejudicial o seu efeito sobre a aprendizagem, memória e, inclusive, a perpetuagáo de altos níveis de estresse durante a vida^3^.

Existem evidéncias de que a intervengáo pré-natal poderia modular o neurodesenvolvimento, reduzindo os danos causados pelo estresse^3^. Para isso, seria necessário aumentar a sensagáo de bem- estar e prazer materno, reduzindo o medo e a dor - ou seja, liberando mais ocitocina endógena. A ocitocina, hormonio liberado em grandes quantidades durante o TP, circula pelo organismo promovendo as contragóes uterinas, mas também é responsável por uma gama de fungóes fisiológicas envolvidas na emogáo da alegria, no prazer e no amor^4^.

A ansiedade pode ser um fator relacionado a complicagóes no TP, devido a ativagáo do sistema nervoso simpático a liberagáo de hormonios do estresse^5^, os quais podem inibir as boas conexóes sinápticas, repercutindo na experiéncia de parto. Dor e ansiedade caminham juntas, uma vez que a primeira pode provocar a contragáo do assoalho pélvico e dos músculos perineais, aumentando a sensagáo de dor no TP^6^. Quando a dor se torna incontrolável, as habilidades de enfrentamento se reduzem, elevando a ansiedade^7^^,^^8^. Logo, a aplicagáo de alternativas para reduzir a ansiedade no processo parturitivo pode ser eficaz no manejo da dor.

O TP e o nascimento sáo eventos amplos e complexos, influenciados náo somente por fatores anatomicos e fisiológicos, mas também pelas emogóes, os sentimentos, a história e as subjetividades de cada mulher. Por isso, é fundamental considerar todos estes aspectos no cuidado obstétrico.

Robbie Davis-Floyd, cientista e antropóloga, estudou obstetrizes nos Estados Unidos da América que se definiam como holísticas por trabalharem com a ‘energia do nascimento', ou seja, a primeira linha de atendimento é no nível energético, redirecionando a energia por meio da mais rica variedade de abordagens, incluindo mente, corpo, emogóes, espírito e ambiente da pessoa no processo de cura, com o uso de formas de medicina e tratamentos que atuam energéticamente^9^.

Nesse paradigma de cuidado holístico, que considera o corpo como campo energético interligado com outros sistemas de energía^9^, é possível realizar a individualizagáo do cuidado a mulher, e reconhecer a importancia de responder as circunstancias particulares durante o TP, pois suas necessidades sáo primordiais no desdobramento de seu processo de parturigáo^9^. A partir dessa perspectiva, observa-se a ampliagáo do conceito de saúde como consequéncia de uma mudanga de padráo e da criagáo de novas políticas de atengáo ao individuo^10^.

No Brasil, foi instituída por meio da Portaria 971 GM/MS, de 3 de maio de 2006, a Política Nacional de Práticas Integrativas e Complementares (PNPIC), que trouxe diretrizes norteadoras para Medicina Tradicional Chinesa e Acupuntura, Homeopatia, Plantas Medicinais e Fitoterapia, Medicina Antroposófica, Termalismo Social e Crenoterapia, no ambito do Sistema Único de Saúde (SUS)^11^.

Em 2018, foram agregadas novas práticas a PNPIC, por meio da Portaria n° 702, de 21 de margo de 2018, abrangendo também a aromaterapia, prática terapéutica que consiste no uso intencional de concentrados voláteis extraídos de vegetais - os óleos essenciais (OE) - para promover ou melhorar a saúde e o bem-estar^12^. Os OE sao extratos provenientes de plantas aromáticas submetidas a processos de destilagao, podendo ser utilizados com fins terapéuticos através das vias cutánea, olfatória, entre otras^13^.

A aromaterapia tem se caracterizado como uma estratégia da medicina complementar utilizada para o alívio da dor no TP, visto que o manejo da dor é um dos objetivos principais do cuidado intraparto nos servigos de obstetrícia^14^. Entretanto, seu crescente uso na assisténcia obstétrica brasileira, em grande medida, nao é guiado por protocolos institucionais, devido a escassez de trabalhos nacionais sobre a temática.

Diante do exposto, defendemos que o desenvolvimento de pesquisas sobre o tema e a elaboragao de protocolos hospitalares, baseados nas evidéncias científicas disponíveis, podem nortear os profissionais que desejam implantar a aromaterapia e aplicar OE no processo parturitivo de forma segura e eficaz. Assim, este estudo teve por objetivos: identificar na literatura científica sobre o uso da aromaterapia e dos óleos essenciais no manejo do trabalho de parto; e elaborar um protocolo hospitalar, a partir dos achados nas publicagóes, sobre aromaterapia e aplicagao de óleos essenciais no trabalho de parto.

## Materiais e Métodos

Trata-se de uma revisao integrativa da literatura^15^, baseada nas seguintes etapas: 1- definigao do tema e selegao da questao de pesquisa, que buscou averiguar de que forma sao utilizados os óleos essenciais e a aromaterapia no manejo do trabalho de parto espontáneo; 2- estabelecimento dos critérios de inclusao e exclusao dos artigos; 3- identificagao dos estudos pré-selecionados e selecionados; 4- categorizagao dos estudos; 5- análise e interpretagao dos resultados; e 6- apresentagao da síntese do conhecimento^16^.

O acronimo 'PICO' foi utilizado para a coleta de dados, sendo 'P - Population', que descreve a populagao estudada: mulheres em TP espontáneo, na fase latente ou ativa; ‘I - Intervention', define as intervengóes testadas pelos estudos: uso de óleos essenciais e/ou aromaterapia; ‘C - Comparison', identifica o grupo controle agregado a intervengao ou investigado: placebo ou nao uso; e ‘O - Outcome' - que sao os desfechos avaliados: redugao da dor no TP, redugao da ansiedade no TP, formas de administragao e efeitos dos OE^17^.

A coleta dos dados foi realizada entre os meses de abril e junho de 2019, nas bases indexadoras de dados Literatura Latino-Americana e do Caribe em Ciéncias da Saúde (LILACS), Cochrane Library e Pubmed. Incluíram-se artigos científicos publicados em periódicos nacionais e internacionais, no período de 2000 a 2019, nos idiomas portugués, espanhol e inglés, que respondessem a questao de pesquisa. Teses, dissertagóes, monografias, livros, artigos de jornais ou revistas sem caráter científico e resumos de pesquisas foram excluídos.

Adotaram-se os seguintes descritores em portugués e espanhol, extraídos dos Descritores em Ciéncias da Saúde (DeCS): aromaterapia, enfermagem, obstetrícia e terapias complementares, aromaterapia, enfermería, obstetricia e terapias complementarias. Também aplicamos termos do Medical Subject Heading (MESH): aromatherapy, nursing, obstetrics e complementary therapies. Para os cruzamentos nos trés idiomas foi utilizado o operador booleano AND.

**Figura 1:**
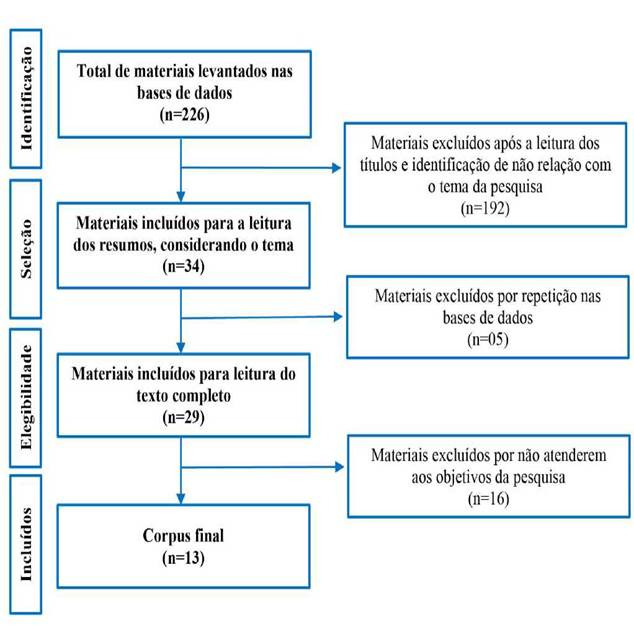
Levantamento dos materiais nas bases de dados.

Artigos indexados em mais de uma base foram contabilizados apenas uma vez. Todo o processo de selegao e levantamento dos dados está exposto na Figura 1.

*Fonte: Karasek G, Mata JAL, Vaccari A; 2019.*

O conteúdo dos achados foi analisado com base na análise temática de conteúdo proposta por Laurence Bardin^18^, em tres etapas: a) pré-análise, na qual desempenhamos a leitura flutuante do material e a organizado dos dados a serem analisados, observando a pergunta e os objetivos da pesquisa; b) explorado do material, com a transformado dos dados brutos em unidades temáticas, por meio de codificado, a partir da identificado das unidades de significado desveladas nos resultados; e c) análise dos conteúdos, envolvendo inferencia e interpretagóes dos achados com base nas referencias adotadas nesta investigado científica. O conjunto de dados deste estudo foi salvo no repositório público Harvard Dataverse^19^.

Visando a atender as normas regulamentadoras de pesquisa e a Lei dos Direitos Autorais número 9.610 de 19 de fevereiro de 1998^20^, todos os dados identificados na literatura foram devidamente referenciados com respeito ao rigor ético e a propriedade intelectual.

Este trabalho foi registrado e aprovado pela Comissao de Pesquisa (COMPESQ) da Escola de Enfermagem da Universidade Federal do Rio Grande do Sul (EENF/UFRGS), em abril de 2019, sob o registro 37003.

## Resultados

Os treze artigos selecionados versaram sobre o uso de óleos essenciais e aromaterapia em mulheres em TP espontaneo. Todos se encontravam no idioma inglés e estavam disponíveis no Pubmed e na Cochrane Library, conforme apresentado no Quadro 1, no qual consta a sinopse dos estudos incluídos, considerando a estratégia PICO e as delimitates apontadas. Destes, oito eram iranianos e os demais tiveram como cenário o Reino Unido.

A maioria^8^^,^^21^^-^^28^ dos trabalhos era do tipo ensaio clínico randomizado (ECR), comparando o uso de aromaterapia com placebo. Alguns relataram a impossibilidade de realizar cegamento nas amostras, em razao da natureza das intervengóes^22^^,^^25^^-^^28^.

Salientamos que nao houve materiais na LILACS que atendessem aos critérios de inclusao desta pesquisa.

**Quadro 1:**
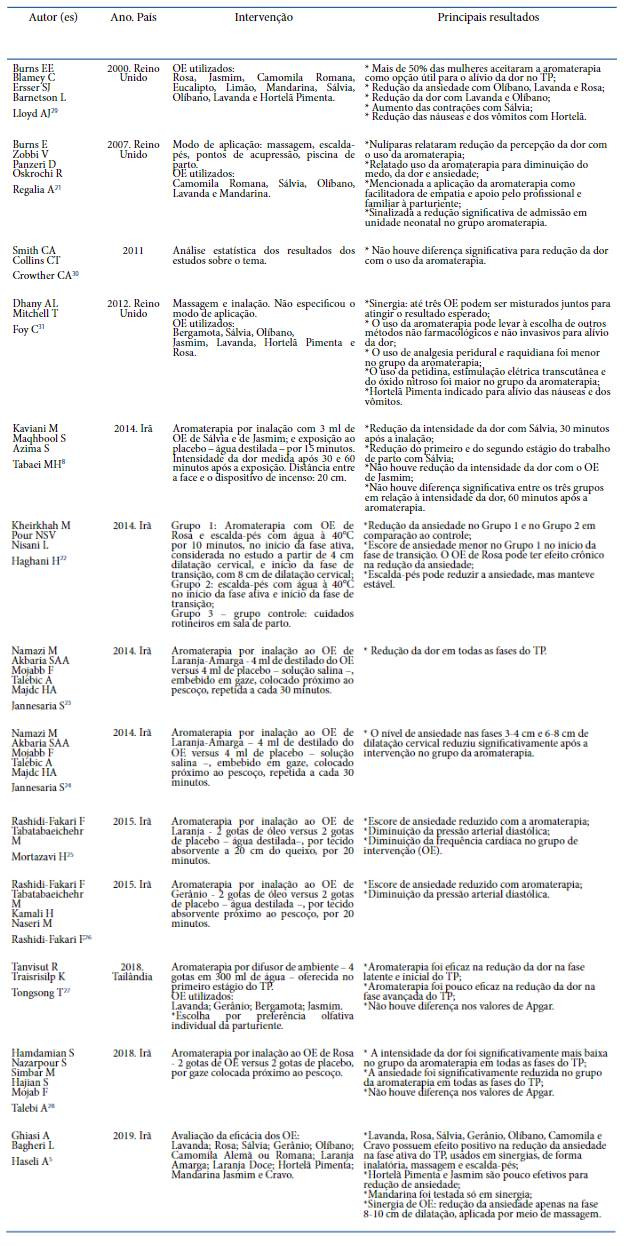
Sinopse dos estudos incluídos na pesquisa com base na estratégia PICO

Fonte: Karasek G, Mata JAL, Vaccari A; 2019.

Os ensaios clínicos randomizados tiveram como cenário o ambiente Hospitalar^8^^,^^21^^-^^28^ e como amostra principal mulheres nulíparas, na fase ativa do TP, gestagáo a termo e classificadas como risco habitual. Apenas um ECR incluiu em sua amostra multíparas^21^. No entanto, os outros tipos de pesquisas incluidas neste trabalho tiveram como populagáo todas as mulheres em TP dos cenários de coleta, indiferentemente da paridade^29^^-^^31^.

Os efeitos da aromaterapia foram avaliados nas diferentes fases do TP. As investigates que abordaram sobre o uso da aromaterapia na redugáo da dor no TP utilizaram a escala visual analógica de dor (EVA), a escala Likert ou uma escala numérica para medir a intensidade da dor antes e após a intervengo com os OE ^8^^,^^21^^,^^23^^,^^27^. Já os artigos que tiveram como propósito estudar o uso da aromaterapia na redugáo da ansiedade, utilizaram principalmente a medida pelo Spielberger's State-Trait Anxiety Inventory (STAI) e a Escala Visual Analógica para Ansiedade (EVAA)^22^^,^^24^^,^^25^^,^^26^^,^^28^.

Os OE estudados e seus respectivos nomes botánicos incluíram Camomila Alemá (Matricaria recutita) ou Romana (Chamaemelum nobile), Cravo (Eugenia caryophyllus), Eucalipto (Eucalyptus globulus), Geránio (Pelargonium graveolens), Hortelá Pimenta (Mentha piperita), Jasmim (Jasminum officinale), Laranja Amarga (Citrus aurantium), Laranja Doce (Citrus sinensis), Lavanda (Lavandula officinalis), Limáo (Citrus limonum), Mandarina (Citrus reticulata), Olíbano (Boswellia sacra), Rosa (Rosa damascena) e Sálvia (Sálvia sclarea).

Quanto a administrado dos OE, em oito estudos foi adotada a aromaterapia por inalado - incluindo difusor de ambiente^8^^,^^23^^-^^28^^,^^31^, dois utilizaram o método de massagem^21^^,^^31^ e dois aplicaram o escalda- pés^21^^-^^22^. Alguns adotaram mais de um método de aplicado da aromaterapia em sua intervendo. Outras modalidades como o uso de piscina de parto e pontos de acupressáo foram abordados em apenas um trabalho^21^.

Tres pesquisas eram revisáo, sendo uma sistemática^5^, uma descritiva-retrospectiva^31^ e uma metanálise^30^. Essas tiveram como propósito mostrar a possível eficácia dos OE na redudo da dor e da ansiedade no TP a partir da percepgáo das parturientes.

Uma investigado científica descritiva e retrospectiva^31^ comparou o uso dos OE de Bergamota, Sálvia, Olíbano, Jasmim, Lavanda, Hortelá Pimenta e Rosa no TP, por meio de massagem e inalado, com mulheres que náo utilizaram aromaterapia. Concluiu que até tres OE podem ser utilizados em forma de ‘sinergia', ou seja, podem ser misturados juntos para atingir o resultado esperado^31^. Além disso, levantou a hipótese de que o uso da aromaterapia pode levar a escolha de outros métodos náo farmacológicos ou menos invasivos para alivio da dor, como, por exemplo, a estimulado elétrica transcutánea, o óxido nitroso e analgésico endovenoso, já que o uso dessas intervengóes foi maior no grupo da aromaterapia, em comparado ao uso de analgesia peridural e raquidiana, que foi maior no grupo controle^31^.

Um estudo de coorte prospectivo^29^ teve como proposta investigar o uso da aromaterapia no TP na prática obstétrica, por meio da aplicagáo de um questionário as parturientes. Mais de 50% das mulheres aceitaram a aromaterapia como opgáo útil para alívio da dor no TP, principalmente com os OE de Lavanda e Olíbano^29^. Outro trabalho^27^ indicou que o escore de dor nas fases latente e inicial do TP foi significativamente reduzido pela aromaterapia, mas náo se mostrou eficaz na fase tardia, quando a dor pode ser mais intensa.

Uma investigagáo de Burns e colaboradores^21^ verificou que a aromaterapia parecia diminuir a percepgáo da dor para nulíparas e estabilizá-la para multíparas. Contudo, Tansuvit, Traisrisilp e TongSong^27^ constataram em uma revisáo que a paridade pode ser um fator de confusáo nos estudos dos OE, quando incluídas somente nulíparas nas amostras, já que a evolugáo das fases do TP entre nulíparas e multíparas pode ser diferente.

Em relagáo aos desfechos neonatais, foi comum entre os estudos que a aplicagáo da aromaterapia e dos óleos essenciais no TP náo alterou o índice de Apgar e, também, náo parecem ter efeito negativo sobre o feto^8^^,^^21^^-^^28^. Além disso, a aromaterapia náo demonstrou efeitos adversos. Ela demanda a presenta do cuidador por mais tempo e revela-se uma facilitadora de empatia e do apoio profissional e familiar a parturiente^21^.

**Figura 2:**
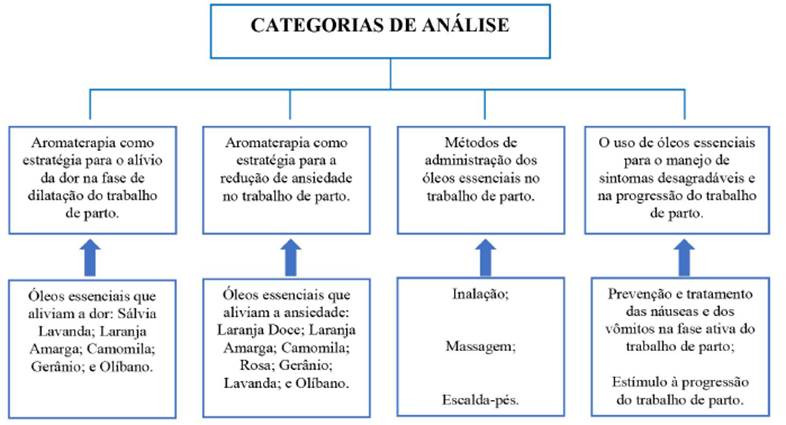
Categorias de análise do estudo

Na análise dos artigos, identificamos as unidades de significado a partir de sua frequéncia de aparigáo nos textos e do conteúdo, delimitando sete subcategorias que culminaram nas seguintes categorias de análise (Figura 2): 1- Aromaterapia como estratégia para o alivio da dor na fase de dilatagáo do trabalho de parto; 2- Aromaterapia como estratégia para a redugáo de ansiedade no trabalho de parto; 3- Métodos de administrado dos óleos essenciais no trabalho de parto; e, 4- O uso de óleos essenciais para o manejo de sintomas desagradáveis e na progressáo do trabalho de parto.

Fonte: Karasek G, Mata JAL, Vaccari A; 2019.

## Discussao

Nesta pesquisa, pudemos constatar que os estudos sobre o uso dos óleos essenciais e da aromaterapia no TP abordam basicamente dois aspectos principais: o alivio ou redugáo da dor e a diminuido da ansiedade. Além destes beneficios, identificou-se: o alivio ou redudo das náuseas e dos vómitos e a estimulado das contrades uterinas no processo parturitivo. Tais dados contribuiram para a elaborado do protocolo hospitalar proposto no final deste trabalho.

A aplicado dos OE pode favorecer a presenta do cuidador por mais tempo, fomentando o desenvolvimento de um relacionamento empático e de apoio capaz de se estender inclusive para o familiar/acompanhante, que se sente mais ativamente envolvido no cuidado, e pode aplicar, por exemplo, uma massagem relaxante com OE^21^.

A seguir, nos aprofundamos na discussáo considerando as categorias de análise emergidas desta investigado. A análise e interpretado do material coletado também se fundamentou em livros emanuais que abordavam sobre os mecanismos de agáo e a aplicagáo dos OE, que contribuíram de forma significativa para elucidar os beneficios da prática da aromaterapia e para a construyo do protocolo apresentado.

### Aromaterapia como estratégia para o alivio da dor na fase de dilatado do trabalho de parto

A dor no TP é sempre subjetiva, sendo significada por cada mulher de acordo com as suas percepgóes, emogóes, história e vivencias. Sendo assim, um dos aspectos mais importantes sobre o uso da aromaterapia é sua atuagáo no sistema límbico. O aroma exalado pelos OE ativa células nervosas olfativas, e, dependendo do tipo de aroma, diferentes neurotransmissores sáo liberados, podendo ocorrer a diminuigáo dos hormonios do estresse e o aumento da secregáo de beta endorfinas, resultando em redugáo no nível de ansiedade e, consequentemente, na percepgáo da dor^29^^,^^37^^,^^38^. Vale salientar que o conceito de dor e a sua percepgáo dependem da interagáo de fatores mentais, físicos e espirituais^32^^,^^33^.

Os OE possuem uma composigáo química complexa, podendo ter de 20 a 200 substancias derivadas principalmente da classe dos terpenos^13^. Ao aplicar o OE, por meio da inalagáo ou da via cutanea, algumas moléculas terpenicas ocupam os nociceptores, estimulando-os e excitando-os a um nível em que eles se tornam dessensibilizados, atenuando fortemente a dor. Estes óleos agem localmente contra a dor e também no sistema nervoso central^13^. Pode-se dizer, entáo, que a aromaterapia é uma estratégia alternativa e útil para controle da dor, além de possuir baixo custo quando comparada a outros métodos e ser de aplicagáo fácil e pouco invasiva^23^^,^^31^.

Os aromas sáo facilitadores de memórias. Ao estimular as lembrangas, permitem que as pessoas revejam detalhes de eventos e emogóes passadas e podem afetar a intensidade da percepgáo da dor^34^. Por isso, é necessário investigar se o OE remete a alguma lembranga positiva ou negativa, para indicar ou náo a sua aplicagáo. Muitas memórias olfativas positivas podem ser proporcionadas pelo aroma, o que harmoniza o ambiente e melhora a conexáo da parturiente com seu processo de entrega ao TP.

Pode haver variagáo na eficácia dos resultados da aplicagáo dos OE nas diferentes fases do TP, sendo influenciada pelo tipo de óleo, método de intervengáo ou fase do processo parturitivo em que é feita^22^. Por exemplo, no estudo de Dhany e colaboradores^31^, a aplicagáo dos OE por meio de massagem reduziu o uso de métodos farmacológicos para o alívio da dor, como analgesia e anestesia no TP, o que, por sua vez, tem o potencial de melhorar os resultados maternos e neonatais^31^. Em contrapartida, tal estudo também observou que o uso da petidina, estimulagáo elétrica transcutanea e do óxido nitroso, foi maior no grupo da aromaterapia, langando a hipótese de que as mulheres desse grupo optaram por métodos pouco invasivos para alívio da dor^31^. A administragáo inalatória também pode ter efeitos benéficos, pois as essencias oleosas acessam o sistema olfativo através de neurotransmissores nas glandulas olfatórias e no sistema límbico, capazes de reduzir a percepgáo da dor e aumentar as emogóes positivas^22^.

Os OE possuem múltiplas fungóes e podem ser utilizados com diferentes finalidades, inclusive variando na forma de implementagáo. Uma pesquisa^8^ verificou a redugáo da intensidade da dor com o uso de Sálvia, 30 minutos após a inalagáo, bem como a redugáo do primeiro e do segundo estágio do TP. No entanto, náo houve diferenga significativa em relagáo a intensidade da dor 60 minutos após a administragáo do OE.

Namazi e colaboradores^23^ avaliaram a intensidade da dor após a inalagáo de OE e observaram que o óleo de Laranja Amarga é útil na redugáo da dor em todas as fases do TP. Já Tansuvit, Traisrisilp e Tongsong^27^, que aplicaram a aromaterapia por difusor de ambiente, concluíram que os OE de Lavanda, Geranio, Bergamota e Jasmim sáo eficazes na redugáo da dor na fase latente e inicial do TP, mas que o método se mostrou pouco eficaz na fase avangada da parturigáo. Estudo^29^ já havia demostrado que a aromaterapia foi aceita como opgáo útil para alívio da dor em 50% das parturientes voluntárias, principalmente com Lavanda e Olíbano. Diante do exposto, defendemos o uso da aromaterapia e dos OE como prática integrativa e complementar no manejo da dor no TP, tanto na fase latente como ativa, incluindo tal recomendado no protocolo hospitalar desenvolvido.

### Aromaterapia como estratégia para a redudo de ansiedade no trabalho de parto

É comum que durante o TP o nível de ansiedade aumente significativamente, principalmente entre as mulheres nulíparas, devido ao medo do desconhecido. A literatura evidencia que em resposta a ativagáo do sistema nervoso simpático ocorre a liberado de adrenalina, noradrenalina e cortisol, o que pode levar a diminuido das contragóes uterinas efetivas, o prolongamento do primeiro e do segundo estágio do parto e até o aumento das intervengóes invasivas, incluindo cirurgia cesariana^5^^,^^25^.

Técnicas de relaxamento, métodos de respiragáo específicos, ouvir música e a medicina complementar sáo práticas náo farmacológicas e náo invasivas que tém sido utilizadas para reduzir a ansiedade durante a parturigáo, promover tranquilidade, conforto, prazer e fomentar emogóes positivas^26^.

A ansiedade no TP é considerada um dos fatores que mais influenciam a intensidade da percepgáo da dor. Quando exacerbada, a ansiedade pode causar a contragáo grave do assoalho pélvico e dos músculos perineais, contribuindo para a sensagáo física da dor^6^. O medo do desconhecido leva a excitagáo simpática, produzindo tensáo nas fibras circulares do útero e rigidez na abertura do colo uterino, repercutindo em tensáo dentro da cavidade^35^^,^^36^. Seu incremento gera uma percepgáo elevada da dor devido a liberagáo de hormonios do estresse em ciclo vicioso^7^^,^^22^. Sendo, assim, é inegável a relagáo da ansiedade com a dor no TP, sendo fundamental a adogáo de práticas no cuidado que favoregam o seu alívio, quebrando o ciclo medo-tensáo-dor, que repercute na satisfagáo e experiéncia materna e familiar.

Uma revisáo sistemática^5^ mostrou que os OE de Lavanda, Rosa, Sálvia, Geranio, Olíbano, Camomila e Cravo possuem um efeito positivo na redugáo da ansiedade na fase ativa do TP, como já havia sido mencionado em estudo de Burns e colaboradores^29^. No estudo de Kheirkhah^22^, o OE de Rosa também foi eficaz na redugáo da ansiedade no início da fase ativa do TP e da fase de transigáo, embora com menor intensidade na última.

Rashidi-Fakari^25^ avaliou a eficácia da aromaterapia com o uso do OE de Laranja no manejo da ansiedade no TP e concluiu que, juntamente com a diminuigáo do escore de ansiedade, reduziu também a pressáo arterial sistólica e a frequéncia cardíaca no grupo da intervengáo. Da mesma forma, em ensaio clínico randomizado sobre os efeitos da inalagáo de Geranio, os parametros fisiológicos foram medidos antes e 20 minutos após a intervengáo; os cientistas concluíram que o OE possui efeitos na redugáo da ansiedade bem como na redugáo da pressáo arterial diastólica^26^. Inclusive, foi observado que o OE de Laranja Amarga pode ser usado tanto para o alívio da dor, como citado anteriormente^23^, como para a redugáo da ansiedade nas fases latente e ativa do TP, por meio da aromaterapia por inalagáo do OE, colocado em gaze e preso próximo ao pescogo das mulheres em TP^24^.

### Métodos de administrado dos óleos essenciais no trabalho de parto

A literatura levantada, apresentada no Quadro 1, traz como os métodos de aplicagáo da aromaterapia mais utilizados no TP a inalagáo, a massagem e o escalda-pés. Sáo métodos simples, exigem poucos recursos e possuem boa aceitagáo pelas parturientes.

Estudos^8^^,^^23^^-^^26^^,^^28^ demonstraram que o uso de uma a trés gotas de OE colocadas em tecido absorvente a uma distancia de 20 centímetros da face, preso a roupa, com inalagáo por 15 a 20 minutos, pode ter o potencial de agir na redugáo da dor e da ansiedade nas diferentes fases do TP. É importante lembrar que para cada indicagáo há um OE específico, o que está evidenciado nos resultados apresentados na segáo anterior. A inalagáo deve ocorrer através de uma inspirado tranquila e profunda, o calor do corpo ou das máos aquecendo a gaze e auxiliando na liberagáo das propriedades do OE.

A massagem tem sido associada a aromaterapia na redugáo dos sintomas desagradáveis que podem ocorrer no TP. Por estimular de forma eficaz a pele, músculos e tecidos, pode gerar sensagáo de relaxamento. Uma investigado científica^31^ referiu o uso da massagem como uma agáo que, além de reduzir a ansiedade das mulheres, ameniza o uso de outros métodos de analgesia no TP. Um dos aspectos mais importantes nesse processo, como já citado anteriormente, é a presenta do cuidador e a sua participado ativa no cuidado a parturiente por meio de massagem^21^.

Para a aplicado da massagem é recomendado que os OE sejam diluidos em 10 mililitros de uma base carreadora, em geral, óleo de semente de uva “Vitis vinífera”^31^. A sinergia pode ser utilizada com diferentes finalidades, por meio de uma mesma aplicado^5^^,^^31^, e a diluid^0^ segura para gestantes é de 1 a 3%^13^. Destacamos que a massagem na regiáo frontal do abdomen é indicada para estimular as contragoes uterinas e deve ser realizada com cautela.

O escalda-pés, ou banho nos pés, foi citado por Kheirkhah e colaboradores^22^ como útil na redugáo da ansiedade no TP. O estudo de Saeki^39^ indicou que o banho com OE de Lavanda por dez minutos é capaz de levar a um equilíbrio do sistema nervoso autonomo, gerando a sensagáo de conforto. Para realizar o escalda-pés, é necessário colocar água a 40 graus Celsius em um recipiente em que caibam os pés da parturiente e diluir o OE em base carreadora, de acordo com a indicagáo desejada^22^. Assim como na massagem, é possível realizar combinagoes de OE para diferentes finalidades.

### O uso de óleos essenciais para o manejo de sintomas desagradáveis e na progressao do trabalho de parto.

Além do potencial para reduzir sintomas desagradáveis, os OE também podem ser utilizados para estimular a progressáo do TP. Isso porque alguns mecanismos de agáo desses óleos mostram-se eficazes na redugáo dos estágios do TP e no fortalecimento do poder uterino^8^.

Uma pesquisa afirmou que a aromaterapia poderia ter o potencial de aumentar as contragoes uterinas em mulheres em TP disfuncional^29^. Observa-se que o manejo adequado da dor proporciona o bem-estar materno e, consequentemente, a progressáo da parturigáo^27^, de forma a relaxar o assoalho pélvico. Ao utilizar a aromaterapia para diminuigáo da ansiedade, impede-se o bloqueio da liberagáo de ocitocina ocasionado pela secregáo aumentada de catecolaminas, que diminuem as contragoes uterinas^31^. Dhany e colaboradores^31^ defendem que a aplicagáo dos OE melhora as contragoes, quando associados a massagem. Evans^40^ descreveu que a aplicagáo dos óleos de Sálvia e Jasmim em gestagoes pós-data podem aumentar o nível de ocitocina e estimular a progressáo no TP, embora seu uso excessivo possa resultar em hipertonicidade uterina; por isso, a importancia da diluigáo adequada.

A literatura científica indica a adogáo do óleo de Hortelá Pimenta na redugáo das náuseas e dos vomitos que ocorrem na fase ativa, abordando sua utilizagáo através de inalagáo na prevengáo e no tratamento desses síntomas^29^^,^^31^. É válido ressaltar que identificamos, em um manual sobre aromaterapia^13^, que náo é indicado o uso do OE de Hortelá Pimenta durante a gestagáo, pois contém cetonas, podendo oferecer riscos na fase de desenvolvimento fetal. Deve ser especificamente utilizado na fase ativa do TP, no manejo das náuseas e vomitos, e náo em continuidade.

### Protocolo hospitalar sobre aromaterapia e aplicado de óleos essenciais no trabalho de parto

Este estudo foi desenvolvido no cerne de um curso de especializado em enfermagem obstétrica - Rede Cegonha, na Universidade Federal do Rio Grande do Sul (UFRGS), localizada em Porto Alegre, Rio Grande do Sul, Brasil. Na ocasiáo, as autoras se preocuparam em desenvolver uma pesquisa quepudesse ser aplicada na prática, contribuindo de forma significativa para o processo de humanizado do parto e nascimento no ámbito do SUS.

Face ao exposto, propos-se, a partir dos resultados e da discussao desenvolvida neste artigo, o protocolo abaixo (Quadro 2), que foi apresentado a uma instituido hospitalar onde uma das autoras atuava. Foi implantado e encontra-se em vigor até o momento.

**Quadro 2:** Protocolo hospitalar sobre aromaterapia e aplicado de óleos essenciais no trabalho de parto

Indicação conforme propósito			
Propósito	Óleo Essencial	Formas de administrado	Frequencia / Fase TP mais indicada
Alívio da dor.	Sálvia; Lavanda; Laranja Amarga; Camomila; Geranio; Olíbano.	Até tres gotas, podendo combinar em siner (uma gota de tres OE diferentes), diluídos em ml de base carreadora; Aplicado por massagem na regiáo lombar; Aplicado por inalacáo.	Pode ser repetida até tres vezes 10 em qualquer fase do TP.
Reducáo da ansiedade.	Laranja Doce; Laranja Amarga; Camomila Romana; Rosa; Geranio; Lavanda; Olíbano.	Até tres gotas, podendo combinar em sinergia Pode ser repetida até tres vezes (uma gota de tres OE diferentes), diluídos em 10 em qualquer fase do TP. ml de base carreadora; Aplicacáo em massagem na regiáo lombar; Aplicacáo por inalacáo.	Pode ser repetida até três vezes em qualquer fase do TP.
Reducáo das náuseas e dos vómitos.	Hortelá Pimenta.	Aplicacáo por inalacáo.	Fase ativa, de 6 a 8 cm de dilatacáo.
Estimular contrates.	Sálvia; Jasmim.	Escalda-pés; Aplicacáo em massagem na regiáo abdominal.	Uma vez na fase inicial ou latente do TP.
Métodos de utilizado dos OE			
	Inalacáo	Escalda-pés	Massagem
Indicares	Fase latente do TP, dor, náuseas.	Edema, relaxamento, estimular contracoes uterinas.	Relaxamento e alívio da dor na regiáo lombar; Estimular contracoes - realizar na regiáo abdominal.
Diluicáo	Sem diluicáo.	Adicionar uma a tres gotas de OE diluídas em 10 ml de base carreadora; Misturar; Diluir em dois litros de água morna a 40°C.	Adicionar uma a tres gotas de OE diluídas em 10 ml de base carreadora; Misturar.
Duracáo	15 a 20 minutos.	Mínimo de dez minutos até 1h.	20 minutos.
Describo da atividade	Convide a parturiente a participar, explicando-a o procedimento e os seus explicando-a o procedimento e seus possíveis efeitos; possíveis efeitos; Prepare a aplicado e pingue uma a tres Prepare a diluicáo; gotas de OE em uma gaze ou algodáo; Escolha a ambiencia adequada - luz, Escolha ambiencia adequada - luz, temperatura, sons, acompanhante; temperatura, sons, acompanhante; Posicione a parturiente de modo que Posicione a parturiente de modo que fique fique confortável; confortável; Marque o horário; Aproxime a gaze, com o OE, ao rosto da Registre em prontuário a prática parturiente, de modo que fique a uma realizada. distancia de 20 cm da face. A gaze pode ser presa a roupa, próxima a regiáo do pescoco; Marque o horário; Registre em prontuário a prática realizada.	Convide a parturiente a participar, explicando-a o procedimento e seus possíveis efeitos; Prepare a diluição; Escolha a ambiência adequada - luz, temperatura, sons, acompanhante; Posicione a parturiente de modo que fique confortável; Marque o horário; Registre em prontuário a prática realizada.	Convide a parturiente a participar, explicando-a o procedimento e seus possíveis efeitos; Prepare a diluicáo; Escolha ambiencia adequada - luz, temperatura, sons, acompanhante; Posicione a parturiente de modo que fique confortável; Realize a massagem na regiáo lombar, pernas, pés e máos, aplicando suavemente a diluicáo na pele da parturiente; Marque o horário; Registre em prontuário a prática realizada.
Descarte	Em rede comum.	Em rede comum.	Em rede comum.
Avaliacáo de efeito	30 minutos após o término da aplicado.	30 minutos após o término da aplicado.	30 minutos após o término da aplicacáo.

Fonte: Karasek G, Mata JAL, Vaccari A; 2019.

## Conclusoes

Por meio desta pesquisa, foi possível realizar uma síntese do conhecimento sobre o uso de OE e da aromaterapia no TP, bem como desenvolver um protocolo hospitalar que pode nortear a adojáo desta PIC na atenjáo obstétrica. Esse é um produto importante para a prática da enfermagem, com possibilidade de aplicajáo e adaptajáo em diferentes cenários.

Predominaram na literatura científica estudos internacionais no idioma inglés, reforjando a inquietado das autoras com a escassez de trabalhos brasileiros que sustentem a aplicajáo dos OE e da aromaterapia no cuidado desempenhado no TP. Destacamos como limitajáo da pesquisa o desenvolvimento da coleta em trés bases de dados, sendo importante a realizajáo de investigares em outras.

Os estudos levantados sugerem a utilizajáo dos OE e da aromaterapia no manejo da dor, da ansiedade e o alivio de sintomas desagradáveis, como náuseas e vómitos. Além disso, a maioria ofereceu informares sobre as formas de administrajáo e os possíveis efeitos, o que colaborou na elaborajáo de um protocolo hospitalar.

A PIC aqui abordada representa uma estratégia náo farmacológica para manejo do TP, de baixo-custo, sem efeitos adversos descritos na literatura investigada, com potencial para colaborar na humanizajáo da atenjáo oferecida as mulheres no processo parturitivo.

Ressaltamos que é importante a realizajáo de novos estudos, sobretudo de campo e nacionais, inclusive sobre os desfechos do protocolo construído neste trabalho, para que o tema ganhe mais corpo e cientificidade para sustentar a prática em obstetrícia.
